# Analysis of acute sinusitis-related early failed implant surface: a combined histological, electron microscopy, and X-ray spectroscopy approach

**DOI:** 10.1186/s40902-022-00346-6

**Published:** 2022-04-26

**Authors:** Truc Thi Hoang Nguyen, Mi Young Eo, Mi Hyun Seo, Soung Min Kim

**Affiliations:** Department of Oral and Maxillofacial Surgery, Dental Research Institute, School of Dentistry, Seoul National University, 101 Daehak-ro, Jongno-gu, Seoul, 03080 South Korea

**Keywords:** Implant failure, Maxillary sinusitis, Osseointegration, Scanning electron microscopy (SEM), Energy-dispersive X-ray spectroscopy (EDS)

## Abstract

**Background:**

Even though dental implants are a reliable choice for dental rehabilitation, implant failures due to various etiologies have been reported. Early implant failures account for 2 to 6% of installed implants and are reported to have a higher rate than late failures, regardless of loading time. We herein report three cases of acute sinusitis and early implant failure with implants that failed within 1 month after installation. The aim of this study was to evaluate the surface properties of early failed implants and peri-implant tissue to determine the early osseointegration pattern in acute sinusitis-related failed implants as well as the possible role of surface contamination in the failure of osseointegration.

**Results:**

A combined histological, electron microscopy, and X-ray spectroscopy approach was used to characterize the surface of non-osseointegrated titanium implants and the surrounding biological tissues. Morphologic scanning electron microscopy revealed a heterogeneous surface and irregular osseointegration. The implant surface was covered mostly by carbon- and oxygen-rich organic matter. Energy-dispersive X-ray spectroscopy surface analysis of three implants showed the incorporation of some contaminants in both the upper and apical regions. Carbon, nitrogen, sodium, silicon, chlorine, sulfur, gold, and zirconium were detected on the surface of one or more failed implants. Fibrosis, lymphocytic, and macrophage infiltrates and a high activation of osteoclasts surrounding the bone graft particles were detected in the surrounding tissues.

**Conclusions:**

The etiology and mechanism of early implant failure, especially in sinus-related cases, as well as the proper management interventions to minimize the rate of early implant failures, are of great concern. No matter how confident and accurate the surgeon’s operation, there may be unknown errors in the whole procedure that no one knows about. Rather than errors related to the implant surface, it is expected that there were invisible problems during the evaluation of the patient’s own unique sinus mucosal inflammation or the operator’s own procedure. Furthermore, well-designed researches are necessary to reveal the effect of material-related factors on acute sinus complication and early implant failure.

## Background

The high predictability of dental implants makes them a reliable choice for dental rehabilitation. This, along with the known long-term success of implant-supported prostheses, has resulted in the wide acceptance of implant therapy by the general population. However, implant failures with various etiologies have also been reported [1]. These failures can be chronologically classified as early and late, respectively, based on a determined time point, such as at the time of abutment connection, at the time of loading, within several weeks after final prosthesis delivery, or during the first year after loading [2–5]. Late dental implant failure is commonly linked to the development of peri-implantitis, excessive loading, and/or inadequate prosthetic construction. Cases of early failure, on the other hand, have been attributed to surgical trauma, poor bone quality and/or quantity, patient medical condition, infection, post-insertion pain, and a lack of primary stability [6].

Early implant failure can be defined as the lack of or inadequate osseointegration or intimate bone-to-implant connection before functional loading. The incidence of early implant failure is reported to be high in specific populations (e.g., patients with implants installed in irradiated jawbones). In general, early implant failure affects approximately 2 to 6% of installed implants [7, 8], and recent studies have reported that the prevalence of implant failure is higher in the early phase than in the late phase, regardless of the loading time [9, 10]. Kim et al. [11] reported that the overall rate of dental implant failure related to sinusitis was 5%. However, to our knowledge, there is no study that has reported the prevalence of implant failure related to acute sinusitis. The etiology and mechanism of acute sinusitis-related early implant failure, as well as the proper management interventions to minimize the rate of early implant failures, are of great concern among clinicians [12]. The vulnerability of osseointegration in the early phase following placement during a decrease in primary stability and an increase in secondary stability is suggested to play an important role in implant early failure.

There are several key factors that must be controlled to support osseointegration of the implants, one of which is the chemical “quality” of the titanium oxide (TiO_2_) superficial layer, including the cleanliness of the implant surface. In addition, the mandatory sterilization method is another aspect to consider, and the validity of some rigorous implant handling protocols is still being questioned. The TiO_2_ thin layer (2–6 nm) has been examined and analyzed in many studies [13]. This TiO_2_ layer is covered by a carbon (C)-dominated contamination layer and traces of nitrogen (N), calcium (Ca), phosphorous (P), chloride (Cl), sulfur (S), sodium (Na), and silicon (Si) [13–16]. It has been hypothesized that surface contaminants may be released from the contaminated implant surface, enhancing and perpetuating the inflammatory response, thus altering the healing process and possibly provoking the dissolution of Titanium (Ti) [15, 16]. Furthermore, in the posterior maxillary region, sinus membrane perforation, metallic particles released during drilling, and displacement of grafting material into the sinus cavity could lead to acute sinusitis, peri-implant inflammation, and subsequent implant failure.

We herein report three cases of acute sinusitis-related early implant failure in which the implants all failed within 1 month after installation. The aim of this study was to evaluate the surface properties of these implants demonstrating early failure and the peri-implant tissue to determine the early osseointegration pattern of failed implants, the potential pathogenesis of acute sinusitis, and the possible role of surface contamination in the failure of osseointegration, using scanning electron microscopy (SEM), energy-dispersive X-ray spectrometer (EDS), and transmission electron microscopy (TEM).

## Methods

### Patient cases

The current study was ethically approved by the Seoul National University Institutional Review Board (S-D20200007) and with the 1964 Helsinki Declaration and its later amendments or comparable ethical standards. A written informed consent was obtained from all of the patients.

#### Case 1: early failed tissue-level implants in the posterior maxilla region

A 68-year-old female patient went to the Oral and Maxillofacial Surgery (OMFS) Department of Seoul National University Dental Hospital (SNUDH) to receive her implant installation at the #26 and #27 positions; the 26th and 27th teeth had been removed 7 months previously due to caries. The patient has a medical history of hypertension with aspirin medication and diabetes mellitus. The patient did not smoke and had good oral hygiene. Following consultation with the surgeon, the patient received two 4.0 × 10-mm tissue-level Stella® implants (Shinhung Co., Seoul, Korea), which were installed with sinus lifting and bone augmentation (Oragraft®; LifeNet Health Co., Virginia Beach, VA, USA) in the #26 and #27 positions.

**Fig. 1:**
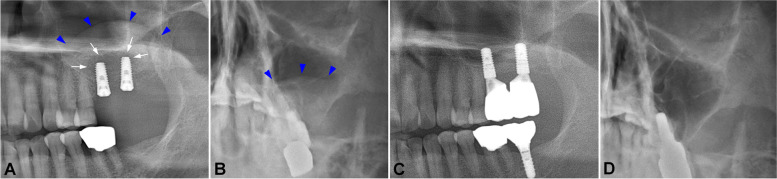
Panoramic view 1 month after implant installation in case 1. There was a radiopaque area in the grafted bone mass on the apical region of the two implants (**A** white arrows). The mucosal thickness, as an image of the pseudocyst, was observed with a panoramic view (**A** blue arrowheads) and Water’s view (**B** blue arrowheads). The panoramic view and Water’s view after implant reinstallation and prosthesis delivery revealed satisfactory bone healing and a clear sinus (**C**, **D**)

One month after the installation, the patient reported pain in the left posterior maxillary region. A clinical examination showed mobility of both implant fixtures. With a panoramic view, resorption of the periapical grafted bone was revealed (Fig. 1A, white arrows). A pseudocyst was also observed in the sinus using Water’s view, around the grafted bone (Fig. 1A, B, blue arrows). Due to the mobility of the implants and sinus-related complications, implant removal was indicated. Incision and open curettage were performed, and the two implant fixtures were removed using forceps. The fixtures were held on the neck carefully to avoid surface damage. The grafted bone was removed, and curettage was performed. The patient reported improvement of her intraoral and sinus symptoms 1 week after the surgery; subsequently, 4 months after implant removal surgery, two 4.1 × 10-mm tissue-level Straumann® implants (Straumann, Basel, Switzerland) were installed at the #26 and #27 positions, together with sinus lifting and bone grafting. The patient experienced uneventful healing upon clinical examination and radiographic examination (Fig. 1C, D). Finally, the prosthesis was delivered and reported to be in good function.

#### Case 2: early failed bone-level implants in the posterior maxilla region

**Fig. 2:**
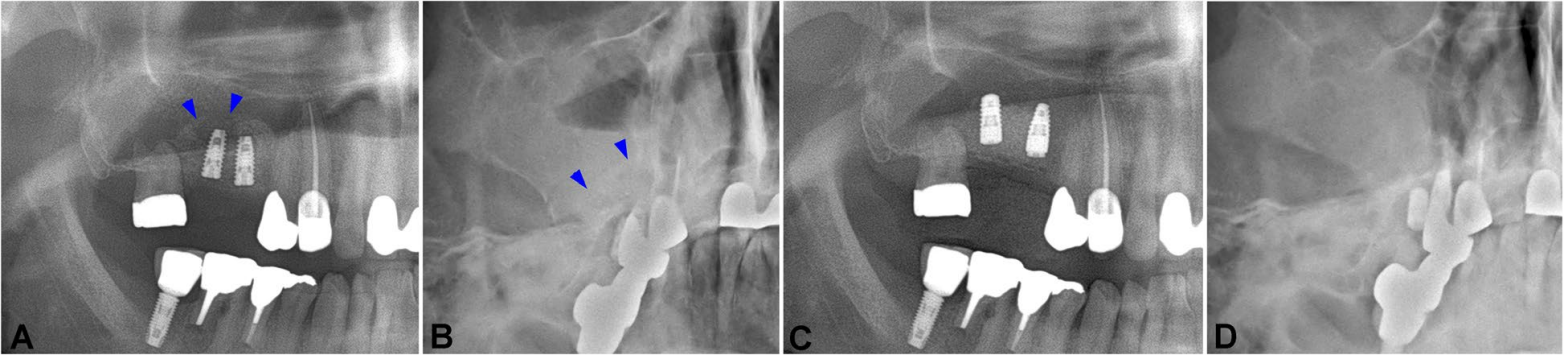
A panoramic view 2 weeks after implant installation in case 2. There was a displacement of grafted bone, and the implant apex was exposed to the sinus cavity (**A** blue arrowheads). The mucosal thickness and fluid level were made apparent using Water’s view (**B** blue arrowheads). The panoramic view and Water’s view after implant reinstallation showed good bone integration and a clear sinus (**C**, **D**)

A 54-year-old male patient presented with a malformed bridge on the right posterior of the maxilla. The patient did not have any specific medical history but had a history of chronic sinusitis and modified endoscopic-assisted sinusitis surgery (MESS) on the left maxillary sinusitis. Noticeably, his alveolar bone height in the maxillary right posterior area was extremely atrophied due to pneumatization of the sinus and prosthesis malfunction. The patient was managed with bridge cutting, sinus lifting, and bone grafting (Oragraft®; LifeNet Health Co., Virginia Beach, VA, USA), and two bone-level 4.0 × 8.5-mm Luna® implants (Shinhung Co., Seoul, Korea) were installed in the #15 and #16 positions. Roughly 2 weeks after surgery, the patient reported the presence of a bad smell and exudate that had begun 10 days after surgery. An intraoral examination confirmed the mobility of both implants. With a panoramic view, displacement of a portion of grafted bone inside the sinus was observed (Fig. 2A, B, blue arrows).

The implants were removed together with all grafted bone, with curettage of the posterior maxillary bone. Sinus irrigation was performed once per week for 3 weeks after the surgery until the sinus symptoms had resolved; however, the biopsy result indicated chronic maxillary sinusitis of the right maxillary sinus. Five months later, two 4.1 × 8.0-mm bone-level Straumann implants were installed at the same position and achieved good stability without any implant or sinus-related complications (Fig. 2C, D).

#### Case 3: early failed bone-level implant in the premolar area

**Fig. 3:**
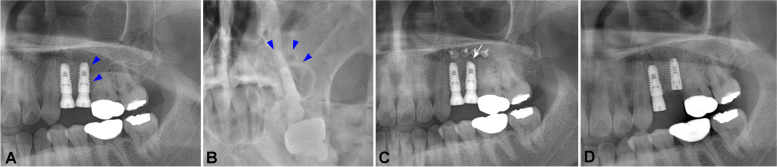
Panoramic view 10 days after implant installation in case 3. There was a radiopaque area observable on the distal side of the #25i implant (**A** blue arrowheads). The mucosal thickness and fluid level were made apparent using Waters’ view (**B** blue arrowheads). A panoramic view taken after the patient underwent implant apex-cutting via MESS (**C** white arrow). The panoramic view after #25i was removed and reinstalled. The implant demonstrated good initial contact with the bone (**D**)

A 30-year-old female patient visited the OMFS Department of SNUDH 10 days after undergoing implant installation and sinus lifting at a local clinic. One week after her surgery, the patient felt pain in the surgery site and discomfort in the nose and sinus; therefore, she visited the Ear, Nose, and Throat Department and was diagnosed with inflamed sinusitis. The patient took antibiotic and anti-inflammation medications daily, but her condition did not improve. When the patient arrived at the OMFS department, she reported pain in the left posterior maxilla, with a bad smell and running exudate in the throat. With a panoramic view, a radiopaque area distal to the #25 implant was noted (Fig. 3A, blue arrows), and there was inflammation observed using Water’s view (Fig. 3B, blue arrows).

The patient was treated with a #25i implant apex using the MESS technique, with the removal of the inflamed tissue in the sinus (Fig. 3C, white arrow). Subsequently, the patient received follow-ups every month, although her sinus symptoms improved and resolved totally after 1 month. Two months after the apex-cutting surgery, the #25i implant exhibited mobility and no improvement in peri-implant bone resorption; as such, 25i implant removal was indicated. The extracted socket was preserved with a bone graft and showed good healing at follow-up visits. Five months after implant removal, one bone-level 3.5 × 10-mm Luna® implant was inserted at the #25 position (Fig. 3D).

### SEM-EDS analysis

**Fig. 4:**
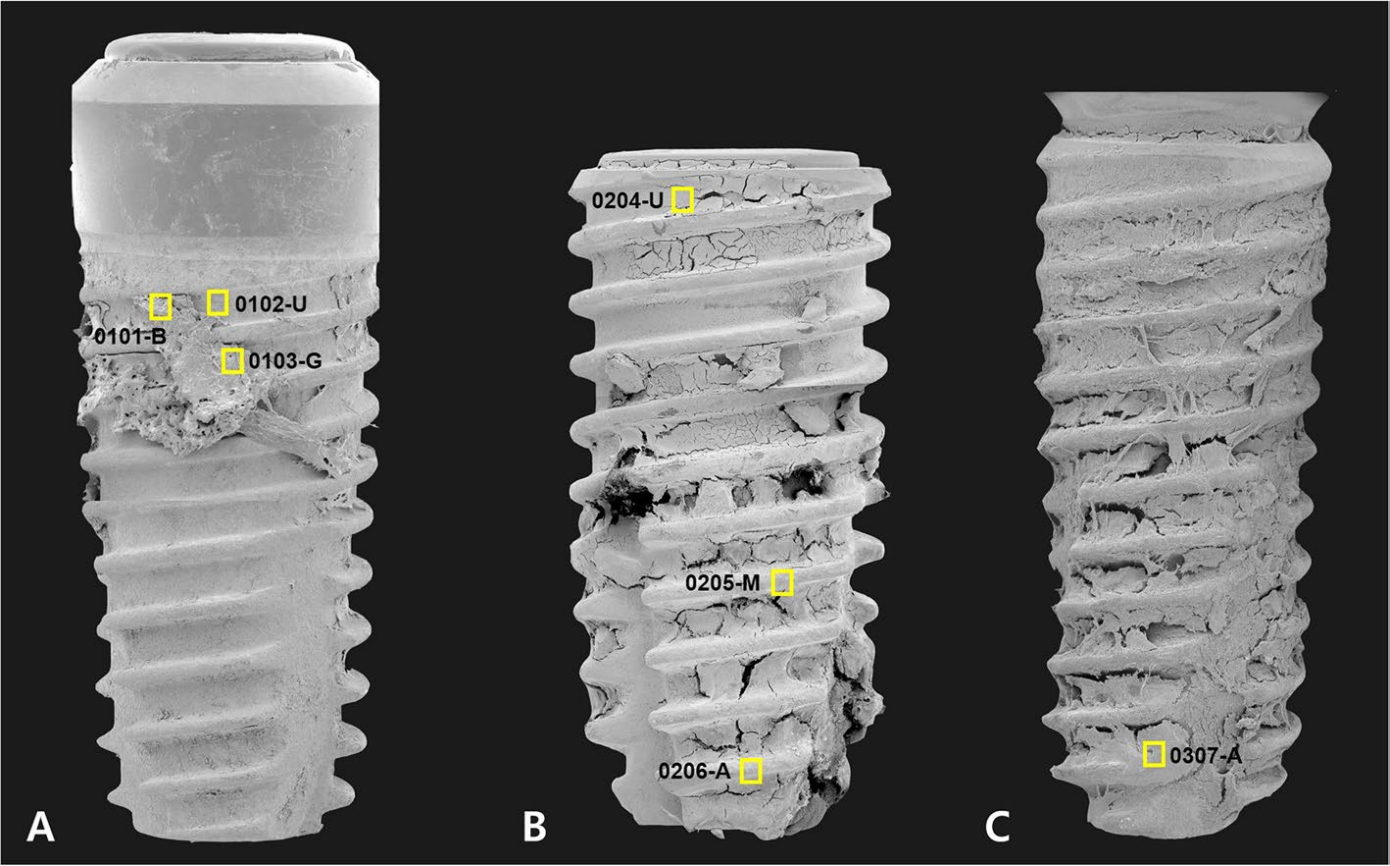
SEM photograph of three fixtures at × 65 magnification with points established by SEM–EDS marked. For fixture no. 01 (**A**), high-magnification SEM photography and EDS analysis were performed at the following three points: integrated bone tissue at the upper region (0101-B), implant surface at the root area of the upper region (0102-U), and grafted bone at the second thread of the upper region (0103-G). For fixture no. 02, the analysis was performed at three points in the upper region (0204-U) and the top of the thread in the middle (0205-M) and apical regions (0206-A). For fixture no. 03, one analysis was performed at the bone tissue in the apical region of the fixture (0307-A)

After removal, the collected implant fixtures and surrounding tissue were immediately placed in a 2.5% glutaraldehyde solution. SEM examination was performed at 10 kV, and low- and high-magnification micrographs were acquired (Apreo S®; Thermo Fisher Scientific, Waltham, MA, USA). The EDS instrument connected to the microscope was an XFlash®6 (Bruker Co., Berlin, Germany) detector, and the ESPRIT® analysis software (Bruker Co., Berlin, Germany) was used to analyze the data. The EDS points were designed as follows: U, implant surface on the upper one-third of the fixture; M, implant surface at the middle one-third of the fixture; B, attached bone tissue on the fixture in the middle region of the fixture; and A, implant surface at the apical one-third of the fixture (Fig. 4). Chemical composition was analyzed under a magnification of × 10,000. The mass concentration (C, wt%) ranges in element concentration achieved from the EDS were classified as follows: major, C greater than 0.1 mass fraction (> 10 wt%); minor, C 0.01 or greater but less than 0.1 mass fraction (1–10 wt%); and trace, C less than 0.01 mass fraction (< 1 wt%).

### TEM and histology analysis

For the TEM examination (JEM-1400 Flash®; Jeol Ltd., Tokyo, Japan), the tissue was stripped into a 1 × 1 × 1-mm block, embedded in epoxy resin, and cut into ultrathin sections (70–80 nm). Sections measuring 1 μm were also stained with Toluidine blue and examined with a light microscope (BX41 Light Microscope®; Olympus Co., Tokyo, Japan). The ultrathin section was thoroughly examined under × 3000 magnification to screen for immune cells. Higher magnifications (× 6000, × 10,000) were used for the examination of cell organelles and metal particles.

## Results

### SEM-EDS analysis

The morphologic SEM analysis of early failed implants revealed a heterogeneous surface and a low rate of osseointegration. All three implant fixtures had attached bone tissue. However, unlike the fixtures from cases 2 and 3, that from case 1 only had the attached bone tissue on the upper part of the implant. There was no detection of bacteria or other infectious organisms on all three implant surfaces. In the case with primary bone formation, “distant” osseointegration was observed. Noticeably, on all examined fixture surfaces, an irregular morphology of a sandblasted and acid-etched surface was observed. The implant surface was covered mostly by C- and oxygen (O)-rich organic matter. The SEM–EDS analysis results of each region of interest are shown in Table 1.

**Table 1:** EDS results of the three cases of early implant failures

Implant no.	EDS-examined position	EDS result (C: weight percentage)			Surface morphology (SEM results)
		C ≥ 10%	10% > C ≥ 1%	C < 1%	
01	Bone tissue in the upper region (0101-B)	C: 38.43% O: 32.90% N: 16.08%	Ca: 7.87% Tl: 2.42% Si: 1.90%	S: 0.41%	- Bone tissue showed irregular structure with no presence of cells or bone lacunae.
		- Low level of Ca and no P signal. - High levels of C, N, and O → large portion of organic content. - Noticeable levels of Tl and Si. - Trace S signal.			
	Implant surface at the first thread of the fixture (0102-U)	Ti: 56.31% O: 21.03% C: 16.90%	Ca: 4.16% Si: 1.60%		- SEM image showed an irregular morphology of a sandblasted and acid-etched surface, with signs of oxidation.
		- Titanium surface with a high level of O → oxidized surface. - Minor level of Ca → low osseointegration on the implant surface. - Si signal.			
	Graft material on the upper region (0103-G)	O: 34.26% C: 23.08% Ca: 23.03% N: 17.72%		Na: 0.97% S: 0.94%	- SEM image showed an irregular bone structure with suggested grafting material particles.
		- There was no detection of the P signal. - High levels of N and O suggested that this is calcified organic material. - Trace Na and S signals.			
02	Implant surface in the upper region (0204-U)	C: 35.79% Au: 26.73% O: 15.52%	Ti: 8.98% Si: 6.48% Na: 3.27% Cl: 3.24%		- Heterogeneous surface with organic particles.
		- Noticeable high level of Au. - Low level of Ti but high levels of C and O → large portion of organic matter. - Si, Na, and Cl were detected to a minor degree.			
	Implant surface in the middle region (0205-M)	Ti: 51.85% O: 15.14% Zr: 11.10%	C: 8.71% Ca: 8.12% Au: 3.03% Na: 1.60%	Si: 0.46%	- Heterogeneous surface with micro-fissures. - Presence of a thin bone layer and organic matter.
		- Titanium surface with a high level of O → oxidized surface. - Noticeably high level of Zr. - Detection of Au and Na at a low level. - Trace amount of Si.			
	Implant surface in the apical region (0206-A)	Ti: 71.72%	O: 9.88% Au: 7.99% C: 7.75% Si: 1.27% Na: 1.02%	Ca: 0.36%	- While the EDS result showed the rich Ti content, the SEM image of the surface revealed an irregular morphology with fibers and micro-debris.
		- Rich of Ti. - Au was detected to a noticeable degree. - Minor signs of Si and Na. - Trace amount of Ca.			
03	Implant surface in the upper region (0307-U)	Au: 40.11% C: 31.69% Si: 11.86% O: 10.14%	Ti: 4.26% Na: 1.94%		- The fixture’s surface was covered with fibers and heterogeneous organic matter.
		- Significantly high level of Au. - High levels of C and O → large portion of organic matter. - Minor Na signal.			

**Fig. 5:**
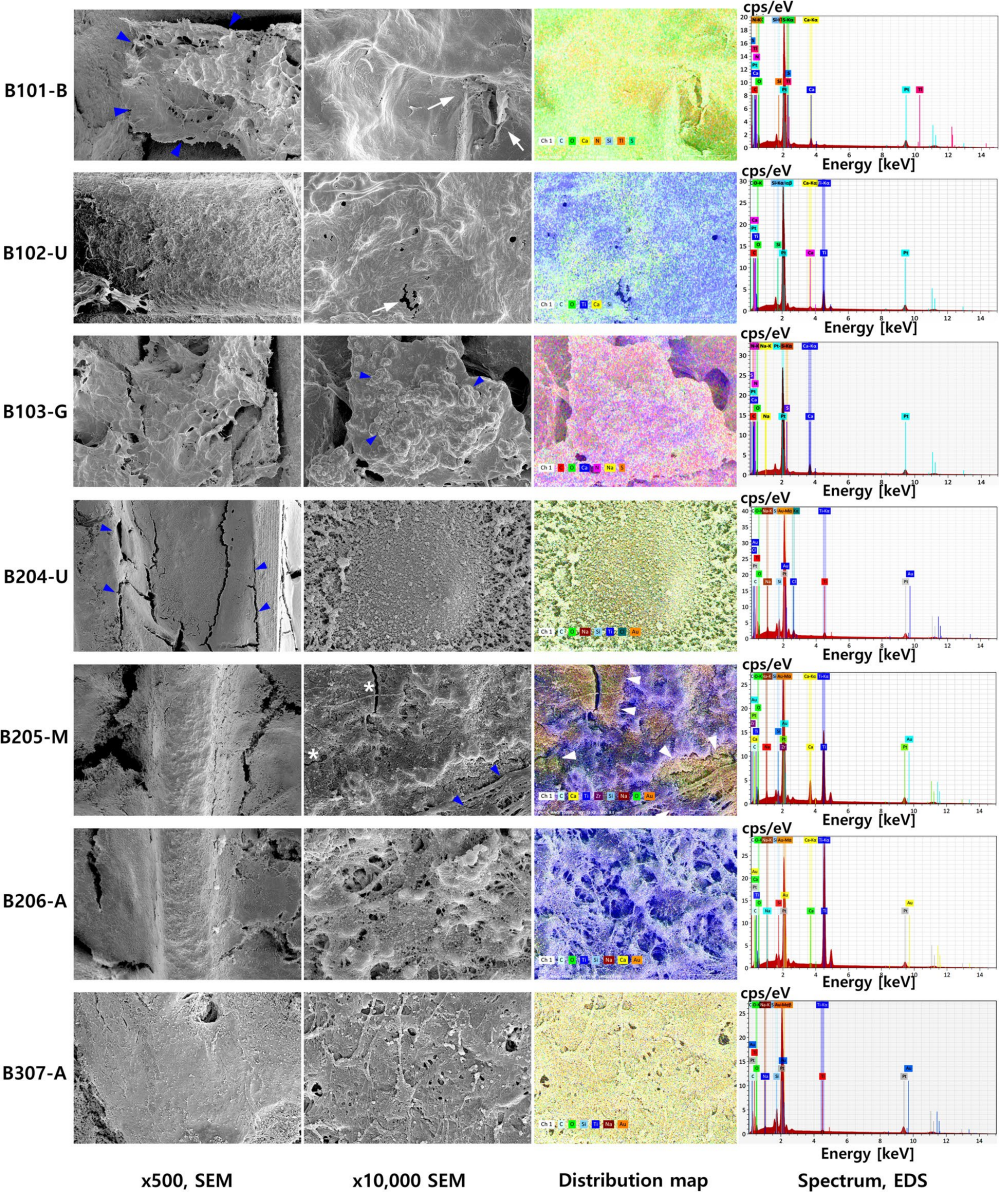
SEM micrographs taken at × 500 and × 10,000 magnifications. An EDS elemental distribution map and a spectrum of representative points pertaining to the three failed implants can be seen. In case 1, the integrated bone on the upper region of the implant presented an irregular structure (0101-B, blue arrowheads). The presence of organic material was observed on the × 10,000 magnification image (0101-B, white arrows) and was confirmed on the elemental distribution map. The implant surface at the root between two threads had an irregular morphology of a sandblasted and acid-etched surface with signs of oxidation. Some micro-fissures were observed (0102-U, white arrow). A sample of bone graft material was examined at 0103-G. The SEM image revealed an irregular bone structure within the sampled grafting material particles (0103-G, blue arrowheads). The Ca signal at this region was high (23.03%); however, there was no detection of P. High levels of N and O suggested that this was calcified organic material. In case 2, “distant” osseointegration was observed (0204-U, blue arrowheads). EDS analysis performed at the upper region (0204-U) revealed a noticeably high level of Au. At the top of the middle thread, a heterogeneous surface with micro-fissures was observed (0205-M, white asterisks). A thin bone layer and organic matter were observed and confirmed on the distribution map. The Zr signal was detected to have the same distribution as the Ti signal. In the thread top of the apical region (0206-A), even though the EDS result suggested rich Ti content, the SEM image of the surface indicated an irregular morphology with fibers and micro-debris. Au was detected at a noticeable level in this region. In case 3, the fixture surface in the apical region (0307-A) was covered with fibers and heterogeneous organic matter. There was no Ca signal and a very high Au signal in this region

In case 1, the integrated bone in the upper region of the implant presented an irregular structure (Fig. 5, 0101-B, blue arrowheads), with no presence of cell or bone lacunae (Fig. 5, 0101-B). However, the presence of organic material was observed at × 10,000 magnification (Fig. 5, 0101-B, white arrows) and was confirmed on the elemental distribution map. There was a low level of Ca, with no phosphate (P) signal, and high levels of C, N, and O, which suggested that this region contained a large portion of organic content. Thallium (Tl) was detected with only a minor signal in this area (2.42%), and Si and S were also detected at low levels (1.90% and 0.41%, respectively). The implant surface at the root between two threads presented an irregular morphology of a sandblasted and acid-etched surface, with signs of oxidation. There was barely any image evidence of the peaks and valleys of the micro-porosity; instead, some micro-fissures were observed (Fig. 5, 0102-U, white arrow). Ti (56.31%), O (21.03%), and C (16.90%) were the major components of this area, suggesting the existence of a high oxidized surface. Ca and Si were recorded to a minor degree in this area (4.16% and 1.60%, respectively). There was a suggestion to examine the bone graft material at 0103-G; SEM imaging revealed an irregular bone structure within the grafting material particles (Fig. 5, 0103-G, blue arrowheads). The Ca signal at this region was high (23.03%); however, there was no detection of P. High levels of N (17.72%) and O suggested that this was calcified organic material.

In case 2, “distant” osseointegration was observed (Fig. 5, 0204-U, blue arrowheads). EDS analysis conducted at the upper region (Fig. 5, 0204-U) revealed noticeably high levels of gold (Au) (26.73%) and Ti (8.98%) ions, while Si, Na, and Cl were recorded to more minor degrees. At the top of the middle thread, a heterogeneous surface with micro-fissures (Fig. 5, 0205-M, white asterisks) was observed, rich with Ti (51.85%) and O (15.14%). A thin bone layer and the presence of organic matter were observed by SEM at a magnification of × 10,000 magnification (Fig. 5, 0205-M, blue arrowheads) and were confirmed on the distribution map. Significant zirconiums (Zr) (11.10%) signal was detected with the same distribution profile as the Ti signal. In the thread top of the apical region (0206-A), even though the EDS results revealed the rich Ti content, SEM imaging of the surface showed irregular morphology with fibers and micro-debris. Au was also detected with a weight percentage of 7.99%.

In case 3, the fixture surface in the apical region (Fig. 5, 0307-A) was covered with fibers and heterogeneous organic matter. There was no Ca signal and a very high Au signal in this region (40.11%), respectively. The presence of a high level of Si (11.86%) in this area was also worth reporting.

### TEM and histology analysis

**Fig. 6:**
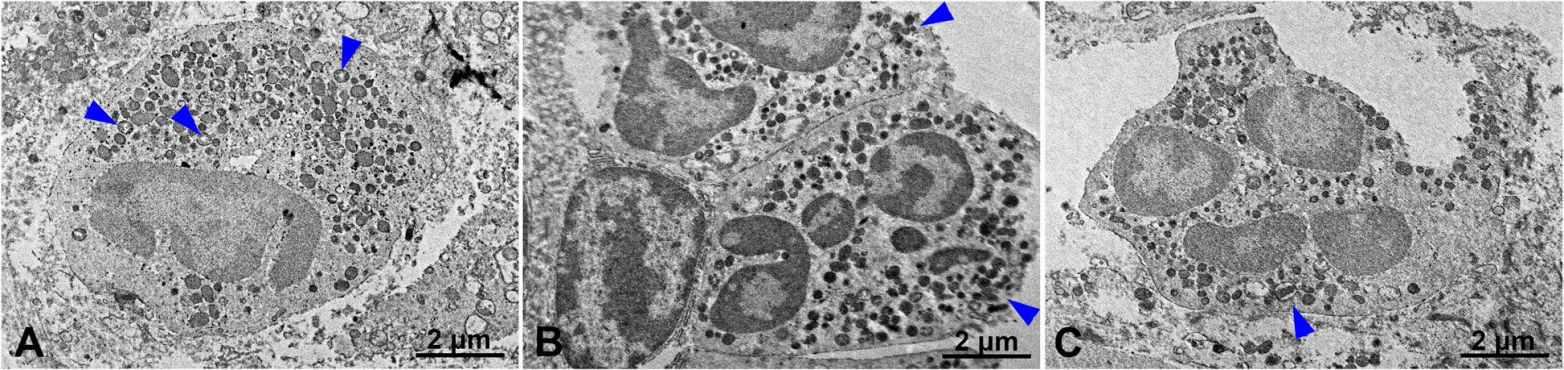
The presence of immune cells was recorded in TEM images of peri-implant soft tissue retrieved from case 1. Images of neutrophils (**A**), macrophages (**B**), and eosinophils (**C**) with cytoplasm filled with exosomes were recorded (**A**–**C** blue arrowheads). There is a sample image of metal particles in the cytoplasm of a macrophage (**A**)

The presence of immune cells was recorded in TEM images of the peri-implant soft tissue retrieved from case 1 (Fig. 6). Images of neutrophils (Fig. 6A), macrophages (Fig. 6B), and eosinophils (Fig. 6C) with cytoplasm filled with exosomes (Fig. 6A–C, arrowheads) were recorded. There was also an indication of the presence of metal particles in the cytoplasm of macrophages (Fig. 6B).

**Fig. 7:**
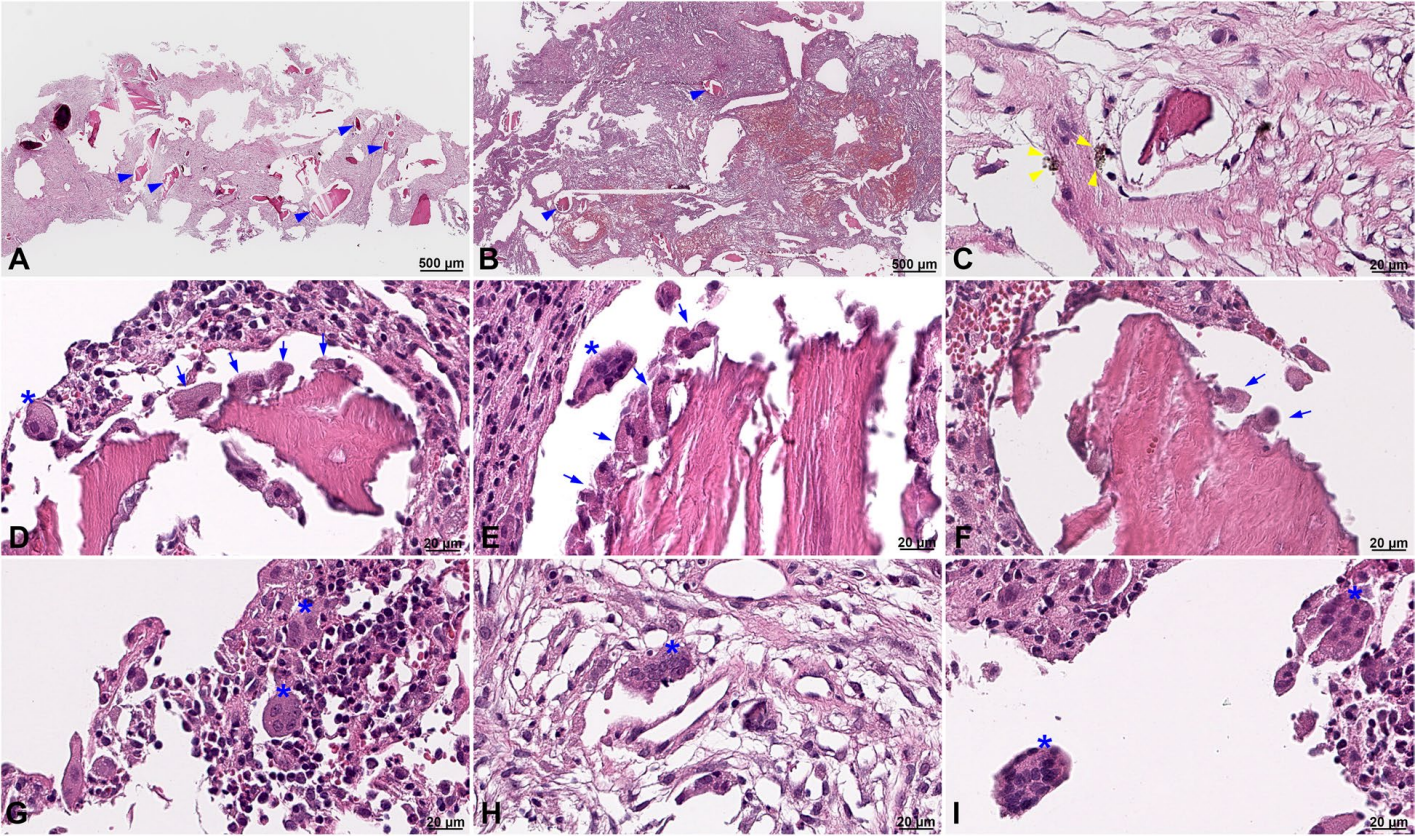
Histological results of peri-implant tissue in case 1. There were bone particles scattered throughout the inflammatory tissue (**A**, **B** blue arrowheads). Metal particles were observed (**C** yellow arrowheads). Osteoclasts were apparent, surrounding the bone particles, supporting the progression of the bone-destruction process (**D**–**F** blue arrows). Multinucleated giant cells were present in the bone particle area as well as scattered in the inflammatory tissue (**D**–**I** blue asterisks)

The histological images obtained from case 1 showed the presence of grafted bone particles scattered throughout the inflammatory tissue (Fig. 7A, B, blue arrowheads). Metal particles were observed in the vicinity of a bone particle (Fig. 7C, yellow arrowheads). Images of osteoclasts surrounding the bone particles were recorded, indicative of the progressing bone-destruction process (Fig. 7D–F, blue arrows). In addition, multinucleated giant cells were present in the bone particle area and scattered throughout the inflammatory tissue (Fig. 7D–I, blue asterisks).

## Discussion

Unlike orthopedic implants placed in an aseptic environment, dental implants are placed in the jaw bone and exposed to the oral cavity, which is a complex biological environment. Therefore, the early failure of implants might be affected by multiple factors. Previous studies have detailed various patient-, surgeon-, and biomaterial-related factors that can play a role in early implant failure [7, 17]; however, the diversity of implant systems has made it is difficult to achieve a consensus about them. The diagnosis and implant removal indication also vary between clinicians. Esposito et al. [6] suggested that early failure is characterized by a lack of osseointegration and the main clinically relevant criterion for the indication of implant removal is the mobility level of an implant. Other subjective signs, such as the patient experiencing pain or sensitivity, signs of infection, and peri-fixture radiolucency, could be improved through time in the healing process and preserving treatment.

Implant placement with sinus lifting and bone grafting is currently a widely used treatment approach in patients with inadequate vertical bone height in the posterior maxillary region to improve the bone quantity and implant osseointegration. Sinus augmentation is highly predictable, with a reported success rate of more than 95% [18]. The survival rate of implants placed in sinuses augmented using the lateral window technique varies between 61.7 and 100%, with an average survival rate of 91.8% [19]. However, several complications may develop, mostly due to disruption of the sinus membrane and displacement of grafting material into the sinus cavity. The incidence of odontogenic maxillary sinusitis after maxillary sinus lifting ranges from 0 to 20%. In the current study, all three cases of implant failure were linked to the sinus-lifting and bone-grafting procedures. Even though the implant demonstrated initial stability after installation, mobility was found after sinus symptoms developed. In cases 1 and 2, resorption and displacement of grafting material were observed. In case 2, a complication was detected 2 weeks after installation, and a change in the air-fluid level in the sinus was observed. Case 1 experienced a longer period of inflammation (one month after installation); therefore, a clearer pseudocyst with a well-defined border was allowed to develop. In case 3, the patient was treated with implant apex-cutting and MESS surgery initially to resolve the sinus irritation and inflammation and to preserve the non-mobile implant. The sinus symptoms were resolved after surgery; however, the peri-implant alveolar bone was subsequently resorbed and the implant failed to osseointegrate. The process of these three cases suggested that patient-related and technique factors could both have played an important role in the initial onset of odontogenic maxillary sinusitis and implant failure. In addition, biomaterial-related factors might have led to implant failure.

According to a meta-analysis study by Kim et al. [11], the only factors that had a significant impact on the rate of postoperative sinusitis were preoperative sinusitis and Schneiderian membrane perforation. Meanwhile, the only factors that affected implant failure were smoking and residual alveolar bone height. The authors found that the implant failure rate was 5.19 times higher when the residual bone height was less than 5 mm [11]. In the current report, the displacement of bone graft material into the sinus cavity was observed in cases 1 and 2, which suggests the presence of sinus membrane perforation and may be the main cause of acute sinusitis. The inflammatory products from acute sinusitis could damage the osteoconduction and bone remodeling of bone graft material and affect the osseointegration of the dental implant, finally leading to implant failure. Moreover, the finding of immune cells under TEM analysis and excessive activity of osteoclasts surrounding the bone particles under the light microscope was evidence of the progressing host reaction response and osteolysis process. In one of our pilot studies that examined the removed peri-implantitis-related implant [20], the SEM and TEM images of the dendritic cells (DCs) were recorded on the implant surface and peri-implant inflamed tissue, respectively. In contrast, there was a dominance of macrophages and the absence of DCs in current cases. These observations gave a glimpse of the role of two antigen-presenting cell populations in the initiate and regulate immune responses. Nevertheless, some studies have reported that sinusitis without chronic change could be treated sufficiently using medication, and there is no certain evidence available proving the direct impact of sinusitis on implant survival [11, 21].

An ideal maxillary sinus bone grafting material should induce a high ratio of vital bone as well as prevent re-pneumonization following resorption of the graft material. Besides autogenous bone, which has been considered as the “golden standard” for bone augmentation, allogenic bone is also used widely in sinus-lifting and bone-augmentation procedures. Allogenic bone graft allows for rapid bone formation and remodeling; however, there are reports of unpredictable bone resorption, and the physical strength of the new bone tends to be weak [22, 23]. The bone-formation rate following allogenic bone grafting is low because the allograft has no osteogenesis and weak osteoinductivity, and the process of sterilization and storage influences both osteoconductivity and osteoinductivity. In the early stage postoperation, blood clotting allows the colonization of bone particles, and the anatomical structure of the sinus walls facilitates mechanical stability of the grafting mass. However, in cases when the rupture of the sinus membrane is available, an increase in the physiologic intra-sinus air pressure due to hemorrhagic reaction, nose-blowing, or sneezing can cause displacement of the bone graft material into the sinus cavity through membrane perforation [24].

Currently, there are many types of allograft materials with different bone origins and chemical compositions. The Oragraft® bone material, which was used in cases 1 and 2, is a particulate bone graft option combining 70% mineralized ground cortical bone with 30% demineralized ground cortical bone. Khanijou et al. [25] studied the physicochemical and osteogenic properties of different types of bone graft materials, including allograft, xenograft, alloplastic, autogenous bone, and human tooth options. EDS analysis revealed that all grafting materials contain O, C, Ca, P, Na, and magnesium, albeit varying percentages of such. As an example, Oragraft® contains C and O at proportions of 34.02% and 34.39%, respectively, while the Ca and P levels of this material are 21.28% and 9.79%, respectively; notably, the Ca/P ratio of Oragraft® is the highest among different types of bone graft material at 2.17. Khanijou et al. [25] also reported that the Oragraft® bone material has an extensive Ca dissolution at the early stage, with a decreasing trend, while the dissolution of P was consistent over 14 days. In the current study, in case 1, even though the Ca level at the integrated bone graft material on the implant surface was at a normal level (23.03%), the P level was undetectable. Furthermore, there was no detection of P on the surface of the implant in cases 2 and 3, with a low level of Ca. We suggest that the consistent dissolution of Ca and P into the peri-implant environment, combined with the coverage of organic matter on the implant surface—which prevented the contact and deposition of Ca and P from the body fluid to the implant surface—may impact and interrupt the initial osseointegration process.

In a previous study of chronic sinusitis-related implant failure in the late phase, we observed that contamination of potentially toxic elements, microorganism infection, and long perforation of the implant apex into the sinus might play a central role in dental implant failure associated with maxillary sinusitis [26]. In the current cases, on the SEM images, there was no detection of bacteria or other infectious organisms on the surface of all three implants, which suggested a low possibility of a bacterial infection etiology. The contamination of the implant and bone tissue with aluminum (Al), iron (Fe), and mercury (Hg) potentially had an influence on the integration of bone tissue and the health of peri-implant tissue. The exposure of the facial prosthesis and its implant to the external environment may have caused the contamination [27]. Noticeably, the significantly high level of gold (Au) on the implant surface and the trace amounts of Au and titanium (Ti) in the bone tissue were recorded, which might have resulted from instability and micro-movement of the implant-abutment connection over an extended period of time [28].

The implant surface morphologic analysis revealed heterogeneous surfaces and a low rate of osseointegration. “Distant” osseointegration was observed and might have arisen during the implant removal or, more likely, due to the inability of the implant fixture to osseointegrate along with the failure of the alveolar as well as grafted bone. Notably, the sandblasted and acid-etched surface might be modified due to oxidization or mechanical abrasion during insertion. However, given the concern that the implants were inserted into the posterior maxillary bone, mechanical abrasion might not be the main factor. Further investigation of this phenomenon using a larger sample size may be warranted.

Commercially pure Ti (cpTi) of grade 4, which is popularly used for implant fixture fabrication, consists of greater than 99% Ti and has an O content of 0.4%. Contamination of other elements is controlled to a maximum of 0.3% iron, 0.05% N, 0.15% hydrogen, and 0.1% C [29]. EDS surface analysis of the three implants in this study showed the incorporation of some contaminants in both the upper and apical regions. C, N, Na, Si, and Cl were detected on the surfaces of the failed implants. According to Kasemo and Lausmaa [30], there is usually a significantly large C signal and a smaller N signal present in dental implants. In the oxide layer of the implant, the intensity of the O signal might not be representative of the true composition of the TiO_2_ layer. The detection of this unrelated element is not related to impurities in cpTi but instead attributed to the C-, N-, Na-, and Cl-containing molecules that progress to adsorption during preparation procedures. The presence of Si is possibly due to the implant surface treatment process [31]. Olefjord and Hansson [32] suggested that inorganic contaminants might block the sites for the O cathodic reaction and therefore result in the dissolution of Ti.

Noticeably, S was only detected in case 1 among the three cases and only in the bone/organic matter region. This raised the question of possible contamination of the graft material in a way that typically causes inflammation and implant failure. Tl, Au, and Zr were uncommon contaminations of the implant surface. Tl amalgam has been used in low-temperature thermometers, and Tl is thought to make its way into dental amalgam due to the recycling of mercury thermometers. Au can be recovered as a dissolved product from other Au intraoral restorations. However, in the implants of cases 2 and 3, Au was detected at a high level in the apical region of the implant. This phenomenon might require further study to reveal the origin of those Au ions. Zr was only detected in case 2 and was hypothesized to have originated from the intraoral breakdown of restoration components. The influence of metallic contaminants on the dissolution rate of Ti in body fluids has not been evaluated to date. It is suggested that foreign ions on the TiO_2_ surface may catalyze the O reaction and thereby promote the dissolution of Ti [33].

As it is written previously, there are three basic causes of implant failure: the surgeon’s ability, the patient’s unique immunity, and the implant material itself. Of course, the comprehensive ability to integrate these three categories as deciding the whole treatment plan could be considered as the most important factor of implant success. Before discussing the cause of the used implant fixture, it is essential to recall and give feedback on whether there were any mistakes of the operator himself that the operator was not aware of, and whether there was any peculiar characteristic of the patient.

## Conclusions

In the three cases of implants inserted in the posterior maxillary with sinus lifting and bone augmentation reported herein, among various implant- and surgery-related factors, implant surface contamination and graft material-related components played an important role in the early implant failure. No matter how perfect and good a surgeon is, he must first look deeply and honestly to see if he has missed anything in his own method and treatment plan. Further well-designed researches are necessary to reveal the effect of material-related factors on acute sinus complication and early implant failure, due to the limited number of this report.

## Data Availability

Data sharing is not applicable to this article as no data sets were generated or analyzed during the current study.

## Abbreviations

SEM: Scanning electron microscopy
EDS: Energy-dispersive X-ray spectroscopy
TEM: Transmission electron microscopy
C: Carbon
O: Oxygen
N: Nitrogen
Na: Sodium
Si: Silicon
Cl: Chlorine
S: Sulfur
Au: Gold
Zr: Zirconium
Ti: Titanium
TiO_2_: Titanium oxide
P: Phosphorous
OMFS: Oral and maxillofacial surgery
MESS: Modified endoscopic-assisted sinus surgery
DCs: Dendritic cells
cpTi: Commercially pure titanium
Tl: Thallium
SNUDH: Seoul National University Dental Hospital
